# Genome-wide identification and comparison of differentially expressed profiles of miRNAs and lncRNAs with associated ceRNA networks in the gonads of Chinese soft-shelled turtle, *Pelodiscus sinensis*

**DOI:** 10.1186/s12864-020-06826-1

**Published:** 2020-06-29

**Authors:** Xiao Ma, Shuangshuang Cen, Luming Wang, Chao Zhang, Limin Wu, Xue Tian, Qisheng Wu, Xuejun Li, Xiaoqing Wang

**Affiliations:** 1 College of Fisheries, Henan Normal University, Xinxiang, Henan 453007 People’s Republic of China; 2 College of Animal Science and Technology, Hunan Agricultural University, Changsha, Hunan 410128 People’s Republic of China; 3 Fisheries Research Institute of Fujian, Xiamen, Fujian 361000 People’s Republic of China

**Keywords:** Gonad, miRNA, lncRNA, ceRNA

## Abstract

**Background:**

The gonad is the major factor affecting animal reproduction. The regulatory mechanism of the expression of protein-coding genes involved in reproduction still remains to be elucidated. Increasing evidence has shown that ncRNAs play key regulatory roles in gene expression in many life processes. The roles of microRNAs (miRNAs) and long non-coding RNAs (lncRNAs) in reproduction have been investigated in some species. However, the regulatory patterns of miRNA and lncRNA in the sex biased expression of protein coding genes remains to be elucidated. In this study, we performed an integrated analysis of miRNA, messenger RNA (mRNA), and lncRNA expression profiles to explore their regulatory patterns in the female ovary and male testis of *Pelodiscus sinensis*.

**Results:**

We identified 10,446 mature miRNAs, 20,414 mRNAs and 28,500 lncRNAs in the ovaries and testes, and 633 miRNAs, 11,319 mRNAs, and 10,495 lncRNAs showed differential expression. A total of 2814 target genes were identified for miRNAs. The predicted target genes of these differentially expressed (DE) miRNAs and lncRNAs included abundant genes related to reproductive regulation. Furthermore, we found that 189 DEmiRNAs and 5408 DElncRNAs showed sex-specific expression. Of these, 3 DEmiRNAs and 917 DElncRNAs were testis-specific, and 186 DEmiRNAs and 4491 DElncRNAs were ovary-specific. We further constructed complete endogenous lncRNA-miRNA-mRNA networks using bioinformatics, including 103 DEmiRNAs, 636 DEmRNAs, and 1622 DElncRNAs. The target genes for the differentially expressed miRNAs and lncRNAs included abundant genes involved in gonadal development, including *Wt1*, *Creb3l2*, *Gata4*, *Wnt2*, *Nr5a1*, *Hsd17*, *Igf2r*, *H2afz*, *Lin52*, *Trim71*, *Zar1*, and *Jazf1*.

**Conclusions:**

In animals, miRNA and lncRNA as master regulators regulate reproductive processes by controlling the expression of mRNAs. Considering their importance, the identified miRNAs, lncRNAs, and their targets in *P. sinensis* might be useful for studying the molecular processes involved in sexual reproduction and genome editing to produce higher quality aquaculture animals. A thorough understanding of ncRNA-based cellular regulatory networks will aid in the improvement of *P. sinensis* reproductive traits for aquaculture.

## Background

Sexual reproduction is a critical process for most vertebrates. Hormones and genes involve in shaping the reproductive abilities of both sexes throughout their lives [1]. Sex-dependent differences are often exhibited in the growth and size of aquaculture animals displaying sexual dimorphism [2]. Reproduction is an important yet complex biological process in animals, and a comprehensive understanding of the genetic mechanisms underlying reproductive traits, particularly from the genomics perspective. The Chinese soft-shelled turtle (*Pelodiscus sinensis*) is an important freshwater aquaculture species in China. The turtle has a sex-dependent growth pattern, with males showing a significantly larger weight and size, thicker and wider calipash, and lower levels of fat than females [3]. Similar to other reptiles and mammals, the soft-shelled turtle has the ability to store sperm in the ovary [4]. Spermatogenesis, copulation, and ovulation are seasonal and segregational in turtles [4, 5]. Many previous studies have focused on sex determination and differentiation in the turtle. However, to the best of our knowledge, no study has explored the genetic mechanisms underlying the reproductive development of the soft-shelled turtle.

The genomes of different species, from worm to human, show similar numbers of protein-coding genes [6], prompting the notion that many aspects of complex organisms arise from non-protein-coding regions. The transcriptome profiling of non-protein-coding RNAs by next-generation sequencing has been successfully used to investigate transcripts and their expression levels. Non-coding RNAs (ncRNAs) regulate gene expression at transcriptional and post-transcriptional levels. Increasing evidence has highlighted that ncRNAs are involved in reproduction process [7, 8].

Regulatory ncRNAs can be divided in three categories based on transcript size: small (sncRNAs), medium, and long (lncRNAs) [9]. MicroRNAs (miRNAs) are an abundant class of sncRNAs (~ 22 nt long) that negatively regulate gene expression at the messenger RNA (mRNA) level [10]. MiRNAs regulate gene expression at the post-transcriptional level by binding to either perfect or imperfect complementary sequences in the 3′ untranslated regions (UTRs) of targets and triggering either degradation of the targets or inhibit their translation [11]. LncRNAs constitute large and diverse class of transcribed non-protein-coding RNA molecules that are more than 200 nucleotides in length [10]. It is known that lncRNAs influence the up-regulation and down-regulation of expression at the transcriptional and post-transcriptional levels. LncRNAs regulate gene expression by epigenetic modification, transcription, and post-transcription modification via DNA methylation, histone modification, and chromatin remodelling [12]. LncRNAs can also bind the typical classes of transcription factor binding sites enriched in promoters, which regulate gene expression [13].

In non-mammal vertebrate animals, large-scale identification of miRNAs and lncRNAs has been implemented in many species. MiRNAs have been shown to engage in regulating the expression of genes that play key roles in follicular development, granulose cell function, oocyte maturation, and ovary pathophysiology [14, 15]. In non-mammalian animals, miRNAs also play important roles in ovary development [16]. A previous study showed that miR-30 was responsible for maternal mRNA clearance during the embryonic development of zebrafish [17]. MiR-9 could bind to the foxl3 3′ UTR in *Monopterus albus*, which may be involved in the process of oocyte degeneration [18]. In mature *Paralichthys olivaceus* gonads, miR-143 and miR-26a showed sex-biased expression [19]. MiRNA is also critically involved in spermatogenesis in mammals [20, 21].

Studies have provided evidence that lncRNAs regulate the processes of mammalian reproduction, including germ cell specification, sex determination, gonadogenesis, gametogenesis, placentation, and pathologies affecting reproductive tissues [22–24]. Knockout of lncRNAs can cause a partial or complete loss of male fertility in *Drosophila* [25]. In mice, *mrhl* RNA can negatively regulate Wnt signalling and becomes down-regulated upon the meiotic progression of spermatogonial cells [26, 27]. In *Daphnia magna*, lncRNA *Dapalr* can transactivate and maintain *dsx1* expression, which produces males in response to environmental stimuli [23]. In female mammals, lncRNA also plays an important role in fertility. *H9* knockout female mice showed altered folliculogenesis and increased follicular atresia, which might be due to the lack of *H9* decreasing the expression of *Amh* by binding the 3′ UTR of *Amh* mRNA [28].

A large number of ncRNAs have been discovered due to advances in genomics and molecular biology. However, regulation of the reproductive system is complicated. Recently, the mechanism of competing endogenous RNAs (ceRNA) was reported as a specific regulatory pathway of lncRNA, miRNA, and mRNA to explain how they exert their influence on protein levels [29–31]. LncRNAs, as competing endogenous ceRNAs, can indirectly regulate mRNAs by acting as miRNA sponges. Investigations regarding lncRNA–miRNA–mRNA networks provide a better understanding of the role of lncRNA–miRNA interactions in mRNA regulation. This might provide new insights for understanding the endogenous differential expression of mRNA in both sexes.

Although miRNAs and lncRNAs have been shown to regulate mammalian tissue development and reproduction, little is known about their sexual dimorphism in gonads and reproduction in turtle families and other reptiles. In the present study, miRNAs and lncRNAs of the ovary and testis were investigated in *P. sinensis* to explore novel ncRNAs in sexual dimorphism and reproduction. Results of the present study may provide the basis for a better understanding of the roles of miRNAs and lncRNAs in the turtle ovary and testis, leading to exploitation of the mechanisms of reproduction in Chinese soft-shelled turtle.

## Results

### Overview of the sequencing data

**Fig. 1:**
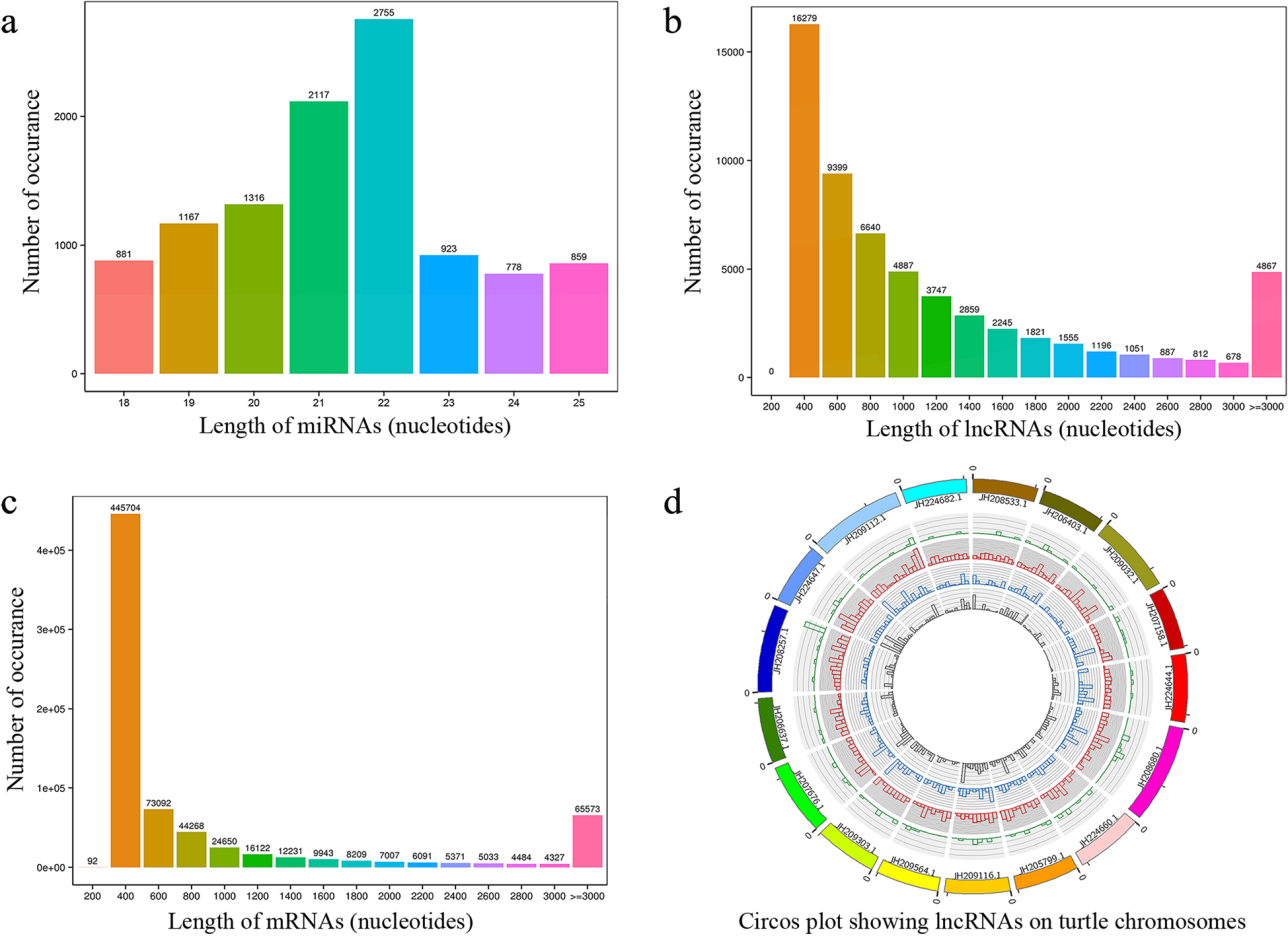
Distribution of miRNAs, mRNAs and lncRNAs in ovaries and testes of Chinese soft-shelled turtle. **a** Length distribution of miRNAs. **b** Length distribution of mRNAs. **c** Length distribution of lncRNAs. **d** Distribution of lncRNAs along the chromosome. (d) The outmost layer of the circos plot is a chromosome map of the turtle genome. The green layer of the circos plot is sense lncRNAs. The red layer of the circos plot is intergenic lncRNAs. The blue layer of the circos plot is intronic lncRNAs. The grey layer of the circos plot is antisense lncRNAs

We constructed cDNA libraries of miRNAs, mRNAs, and lncRNAs using the RNA from the ovaries and testes. After filtering out low-quality transcripts, 5′ and 3′ adapters, and reads < 18 nt, a total of 113.5 M of clean reads was produced by Illumina technology for miRNAs. The 21 and 22 nt length transcripts were the most abundant (Fig. 1a), and 60.4% of high-quality reads were mapped to the turtle genome (Pelsin-1.0, NCBI). We obtained 153.25 Gb of clean reads for mRNA and lncRNA sequencing. The length distributions of lncRNAs and mRNAs are shown in Fig. 1b and c. After mapping the genome, approximately 84.41% ~ 87.72% of the reads were mapped to intergenic regions in the *P. sinensis* reference genome (Fig. 1d, Additional file 1).

### Identification of the differential expression of mRNAs, miRNAs, and lncRNAs

**Fig. 2:**
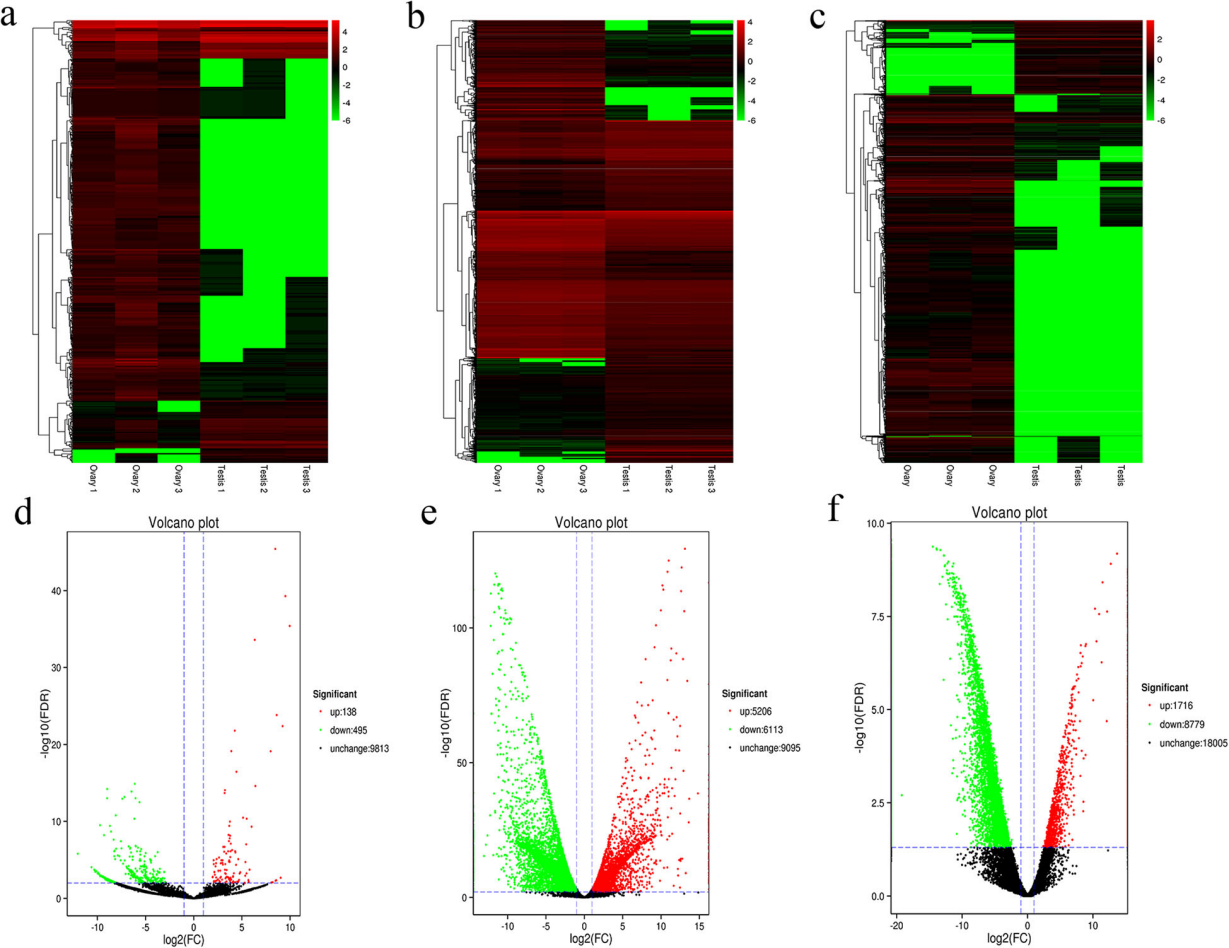
Identifying differentially expressed miRNAs, mRNAs and lncRNAs by transcriptome sequencing in ovaries and testes of Chinese soft-shelled turtle. **a-c** Heatmap of DEmiRNAs, DEmRNAs and DElncRNAs in ovaries and testes. **d-f** Volcano plot of DEmiRNAs, DEmRNAs, and DElncRNAs

According to the miRNA expression profiles, we detected 10,446 novel miRNAs. A total of 633 miRNAs were significantly differentially expressed between the ovaries and testes (*P* < 0.05), including 138 up-regulated miRNAs and 495 down-regulated miRNAs (Fig. 2a, d, Additional file 2). These DEmiRNAs belonged to 438 families (Additional file 3). Among these DEmiRNAs, we identified a set of miRNAs that were reported to regulate animal reproduction, including miR-133, miR-138, miR-145, miR-143, and miR-378.

We detected 20,414 mRNAs, and 11,319 mRNAs were differentially expressed based on sex, including 5206 up-regulated mRNAs and 6113 down-regulated mRNAs (Fig. 2b, e, Additional file 4). A total of 28,500 lncRNAs with 10,495 DElncRNAs were detected, including 1716 up-regulated lncRNAs and 8779 down-regulated lncRNAs between ovaries and testes (Fig. 2c, f, Additional file 5). Among the differentially expressed lncRNAs and miRNAs, 3 miRNAs and 917 lncRNAs exhibited testis-specific expression, and 186 miRNAs and 4491 lncRNAs showed ovary-specific expression. Prediction of the potential targets of lncRNAs in *cis* and *trans* was performed to investigate the function of lncRNAs (Additional file 5). After searching for protein-coding genes 100 kb upstream and downstream, 3904 DElncRNAs were found to correspond to the regulation of protein-coding genes in *cis*. The target genes included *Foxl2*, *Cyp19a1*, *Gper*, *Esr*, *Dazl*, and *Sox30*, which suggests that the reproductive process might be regulated by the action of these lncRNAs on protein-coding genes. Conversely, we identified 2160 lncRNAs showing *trans* action by LncTar, including a set of genes that might regulate reproduction.

### Functional analysis of DEmiRNAs and DElncRNAs

To annotate the molecular functions of the differentially expressed miRNAs, both RNA hybrid and MiRanda software were used to improve the prediction of miRNA targets, resulting in 8088 target genes including 2814 differentially expressed genes that were potentially regulated by 633 DEmiRNAs. GO categories of miRNAs and lncRNAs were assigned to all target genes based on the following three ontologies: cellular component, molecular function, and biological process (Additional files 6, 7, 8). Functions of target genes in the cellular component category mainly focused on cell part, cell, and membrane. Based on molecular function, the most abundant target genes were focused on binding, followed by catalytic activity. Regarding biological process, the most abundant target genes were focused on single organism process, followed by cellular process, and biological regulation.

KEGG pathway enrichment analysis revealed that the DEmiRNAs were involved in 186 signalling pathways (Additional file 9). The identified metabolic networks were related to neuroactive ligand-receptor interaction and regulation of the actin cytoskeleton. The most abundant target genes of DEmiRNAs focused on glyoxylate and dicarboxylate metabolism. We detected at least 13 pathways involved in reproductive biology, including oocyte meiosis, TGF-β signalling, ovarian steroidogenesis, GnRH signalling, Wnt signalling, cAMP signalling, steroid biosynthesis, steroid hormone biosynthesis, MAPK signalling, p53 signalling, RNA polymerase, metabolism of xenobiotics by cytochrome P450, and mTOR signalling.

KEGG pathway enrichment analysis showed that the DElncRNAs were involved in 225 signalling pathways in a *trans*-regulatory manner and 221 signalling pathways in a *cis*-regulatory manner (Additional file 10, 11). The KEGG pathway enrichment analysis revealed that the DElncRNAs were involved in oocyte meiosis, steroid hormone biosynthesis, Wnt signalling pathway, GnRH signalling pathway, p53 signalling pathway, apoptosis, MAPK signalling pathway, AMPK signalling pathway, TGFβ signalling pathway, cAMP signalling pathway, RIG-I-like receptor signalling pathway, mTOR signalling pathway, and insulin signalling pathway.

### Validation of differentially expressed miRNAs and lncRNAs

**Fig. 3:**
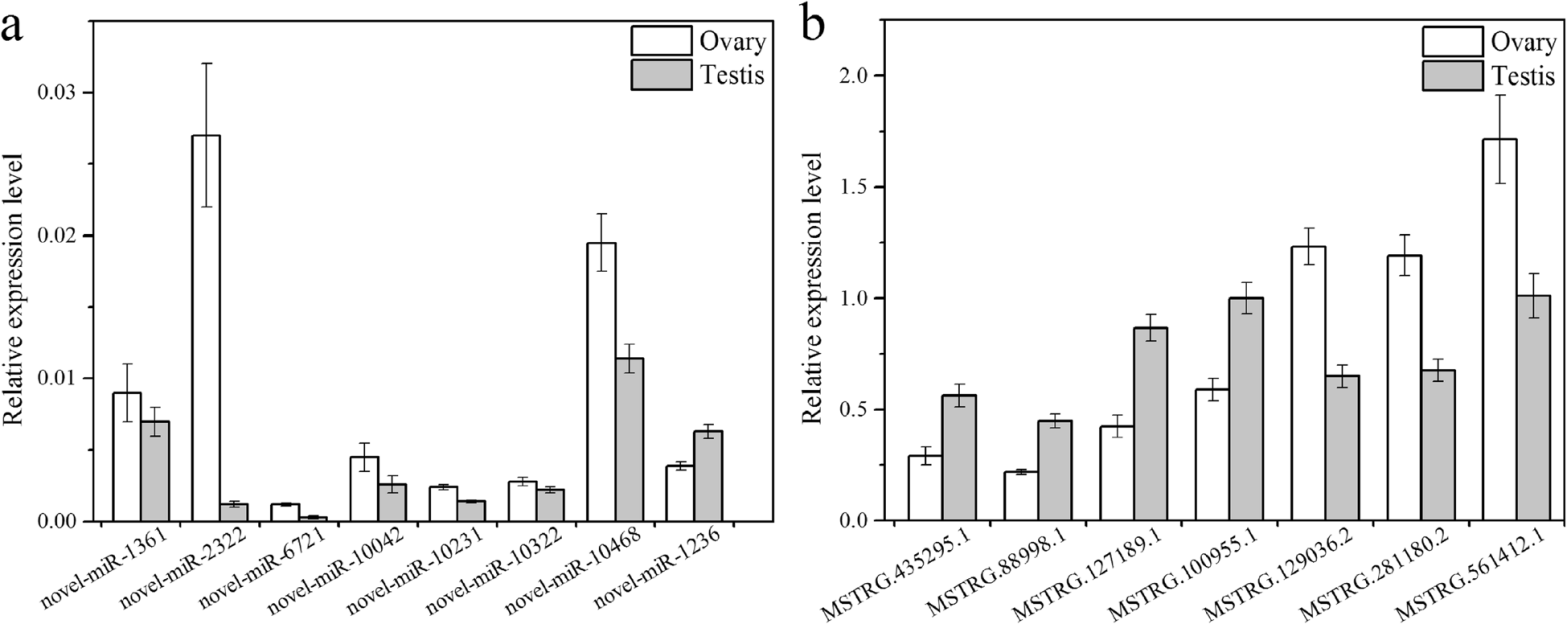
qRT-PCR assays for validating DEmiRNAs (**a**) and DElncRNAs (**b**)

To validate the sequencing data of miRNAs and lncRNAs, ten DEmiRNAs and ten DElncRNAs were randomly selected to test their relative expression in ovaries and testes. The expression of eight miRNAs and seven lncRNAs in ovaries and testes was consistent with the results of RNA sequencing. Among the miRNAs, novel-miR-1361, novel-miR-2322, novel-miR-6721, novel-miR-10,042, novel-miR-10,231, novel-miR-10,322, and novel-miR-10,468 were downregulated in testis, while novel-miR-1236 was upregulated in testes (Fig. 3a). Among the lncRNAs, MSTRG.435295.1, MSTRG.88998.1, MSTRG.127189.1, and MSTRG.100955.1 were upregulated in testes, while MSTRG.129036.2, MSTRG.281180.2, and MSTRG.561412.1 were downregulated in testis (Fig. 3b). The expression patterns of these miRNAs and lncRNAs among different groups were well-matched with the RNA-Seq data, which could guarantee the accuracy of subsequent functional analysis.

### Construction of compete endogenous (ceRNA) networks

**Fig. 4:**
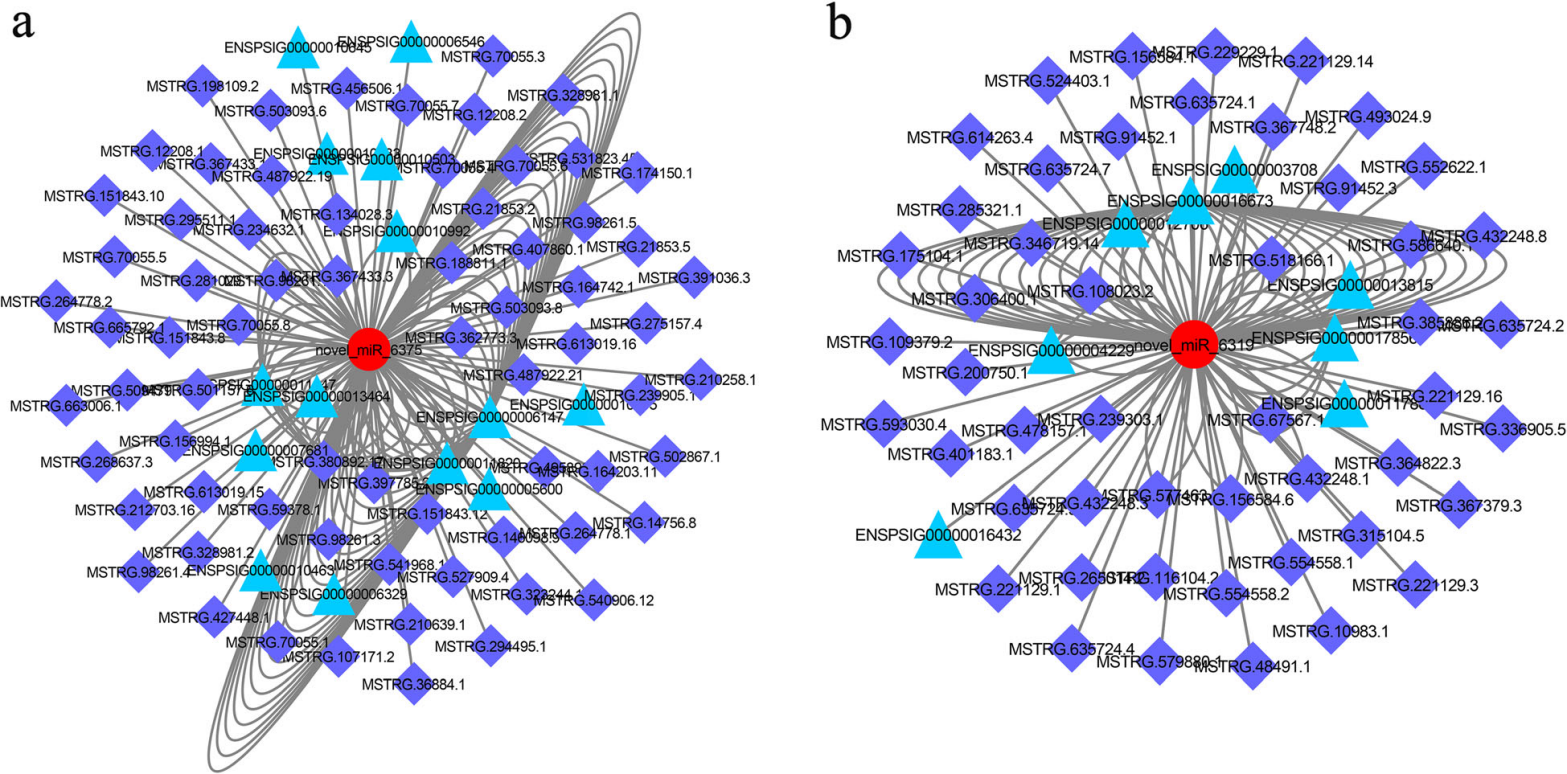
LncRNA-miRNA-mRNA competing endogenous RNA (ceRNA) network of differentially expressed genes in ovaries and testes of Chinese soft-shelled turtle. In the network, red circles = DEmiRNAs, blue triangles = DElncRNAs, and mazarine = DEmRNAs. **a** ceRNA network of novel-miR-6375. **b** ceRNA network of novel-miR-6319

To construct the ceRNA networks, we screened miRNAs that included miRNA response elements, which could bind with both lncRNAs and mRNAs. We constructed a series of ceRNA networks of mRNAs, miRNAs, and lncRNAs related to the DE genes by integrating the expression profiles and regulatory relationships among the mRNAs, lncRNAs, and miRNAs from the high-throughput sequencing data (Additional file 12). These networks included 102 DEmiRNAs, 635 DEmRNAs, and 1621 DElncRNAs. The DEmiRNAs included novel-miR-227, novel-miR-9914, novel-miR-6375, novel-miR-1222, novel-miR-6721, novel-miR-2026, novel-miR-6671, novel-miR-642, novel-miR-6319, and novel-miR-42, etc. These ceRNA networks included a set of mRNAs regulating reproduction (Fig. 4a, b, Additional file 12). For instance, *Dazl* mRNA and MSTRG.71049.8 shared a common binding site of the miRNA novel-miR-1222. We also identified *Wt1*, *CREB3l2*, *Gata4*, *Wnt2*, *Nr5a1*, *Hsd17*, *Igf2r*, *H2afz*, *Lin52*, *Trim71*, *Zar1*, and *Jazf1* in the ceRNA network. These miRNAs and mRNAs participate in regulating the reproductive process, including meiosis and spermatogenesis.

## Discussion

The turtle genome showed a large proportion of non-coding regions, indicating that this part of the genome carried an abundance of untapped information, which needs to be explored. Increasing evidence has shown that miRNA and lncRNA have emerged as regulators in animal reproduction via the control of gene expression [28]. However, their exact functions in the soft-shelled turtle remain poorly understood. Despite limited studies that have identified lncRNAs in the turtle [3], the miRNAs and lncRNAs in the database are still insufficient. In the present study, to understand the molecular mechanism involved in the reproduction of *P. sinensis*, we analysed the genome-wide expression of miRNAs, lncRNAs, and mRNAs in the mature ovaries and testes during the reproductive season. After filtering, we obtained 10,796 miRNAs and 58,923 lncRNAs that were not reported previously in the miRbase and lncRNA databases. The lengths of the miRNAs ranged from 18 to 25 nt. In a previous study, Huang et al. [32] identified only 10 miRNAs in *P. sinensis* based on EST and GSS information using a bioinformatics approach. Zhang et al. [3] identified 5994 lncRNAs by high-throughput sequencing in juvenile turtle gonads. MiRNAs and lncRNAs have been shown to have stage-specific expression in animals [16, 33]. The different developmental stages and the methods utilised in different studies might be responsible for the discrepancies found.

We obtained 633 DEmiRNAs, 11,319 DEmRNAs and 10,495 DElncRNAs. The database included many target genes for miRNAs and lncRNAs that might regulate turtle reproduction, such as *Cyp19a1*, *Gper*, *Esr1/2*, *Sox30*, *Dazl*, and *Foxl2*. A total of 8 miRNAs and 7 lncRNAs were verified using qRT-PCR. Among these miRNAs, 7 miRNAs were upregulated in testis, while 1 miRNA was downregulated. For the lncRNAs, 4 lncRNAs were upregulated, while 3 lncRNAs were downregulated. The qRT-PCR results were well matched to the high-throughput sequencing data.

Mature miRNAs and lncRNAs are crucial for the regulation of gene expression in different ways [34, 35]. GO annotations for the targets were obtained using topGO software. The most abundant differentially expressed genes were involved in single organism process, followed by cellular process and biological regulation, indicating that abundant DEmiRNAs might be involved in the reproductive process and reproduction. The GO analysis of DEmiRNAs and DElncRNAs showed that some terms under the biological process and molecular function categories were related to sex-specific reproduction. In the single organism process, the targets of DEmiRNAs and DElncRNAs included *Cyp19a1*, *Ar*, *Esrrb*, *Catsper2*, and *Pgr*, etc., which were proven to be important for gonadal development, and the results indicated that DEmiRNAs and DElncRNAs might be involved in reproductive regulation.

The DEmiRNAs identified in the soft-shelled turtle belong to 439 families after mapping on the genome, including let-7, miR-10, miR-130, miR-133, miR-138, miR-145, miR-143, miR-202, miR-224, and miR-378. In the majority of cases, the miRNAs and their targets were correlated with animal reproduction [36–39]. MiR-202-3p could regulate human Sertoli cell proliferation, apoptosis, and synthesis functions by targeting *LRP6* and cyclin D1, which belong to the Wnt/β-catenin signalling pathway [40]. Sun et al. [41] reported that miR-378 could indirectly regulate oocyte maturation, possibly via inhibiting oocyte-cumulus apoptosis in mice, and a similar function of miR-378 in porcine was observed [42]. *SMAD5* and *MSK1* were miR-130b targets. In bovine cumulus cells, miR-130b could alter lactate production and cholesterol biosynthesis, and it could inhibit oocyte maturation in vitro by reducing the first polar body extrusion, the proportion of oocytes reaching the metaphase II stage, and mitochondrial activity [43]. MiRNAs are not only involved in ovary development but also involved in testis development and male reproduction. MiRNA-20 and miRNA-106a promote the renewal of spermatogonial stem cells via targeting *Stat3* and *Ccnd1* [39]. In mice, miR-224 promotes spermatogonial stem cell differentiation and self-renewal via targeting *Dmrt1* [44]. Overexpression of miR-224 increased the expression of *GFRα1* and *PLZF* through the downregulation of *Dmrt1*. In the present study, miR-10 and miR-202 expression was significantly higher in the ovaries than the testes; however, miR-133, miR-143, and miR-145 were significantly higher in testes than ovaries. Furthermore, we identified abundant DEmiRNAs whose targets were involved in reproductive regulation, and further functional analysis could be carried out based on the database.

LncRNAs are recognised as important functional regulatory factors in the regulation of eukaryotic gene expression in a variety of biological processes. The function of lncRNAs occurs across a range of animal reproductive processes, including sex determination, meiosis, spermatogenesis, and imprinting, via epigenetic processes including DNA and histone methylation, chromatin looping, and nucleosome positioning [35, 45]. In *Drosophila*, knocking out testis-specific lncRNAs resulted in a partial or complete loss of male fertility [25]. LncRNA *H19* could regulate the IGF-1 signalling pathway, which resulted in regulation of the proliferation and apoptosis of male germline stem cells in bovines [46]. Furthermore, the *H19* imprinting control region could acquire parent-of-origin-dependent methylation after fertilisation independent of the chromosomal integration site or the prerequisite methylation acquisition in male germ cells [47]. LncRNA *THOR* contributed to the mRNA stabilisation activities of *IGF2BP1* and was isolated to spermatocytes during meiosis II, and knockout of *THOR* resulted in fertilisation defects in zebrafish [48]. However, most lncRNAs evolved rapidly and are less conserved, with more than 80% of lncRNA families being of primate origin [49]. In the present study, we identified 28,500 lncRNAs including 10,495 DElncRNAs. Prediction of targets showed that a large number of DElncRNAs might regulate gonadal development, and further investigation should be undertaken to reveal their functions in the turtle.

MicroRNAs are negative regulators of gene expression via decreasing the stability of target RNAs or limiting their translation. Recently, evidence has shown that lncRNAs and mRNAs can bind a miRNA binding site and that miRNA acts as a sponge [29, 50]. In the present study, we constructed lncRNA–miRNA–mRNA networks for sex-specific expression based on high-throughput sequencing data in the turtle. We characterised DEmiRNAs and DElncRNAs by the target mRNA, including *Wt1*, *CREB*, *Gata4*, *Wnt2*, *Nr5a1*, *Hsd17*, *Igf2r*, *H2afz*, *Lin52*, *Trim71*, *Zar1*, and *Jazf1*. The target genes of miRNAs and lncRNAs play important roles in the reproductive processes. *Wt1* regulates Sertoli and granulosa differentiation during gonad development by binding the *Sf-1* promoter [51]. The inhibition of *CREB* could reduce oocyte meiotic resumption and cumulus cell expansion [52]. Deshpande et al. [53] reported that *Wnt2* might stimulate germ cells in male embryos to re-enter the cell cycle. *Nr5a1*/*Sf-1* could bind the *Cyp19a1* promoter, which is crucial for functional maintenance of the ovary [54]. *Zar1* is the first identified oocyte-specific maternal-effect gene that functions at the oocyte-to-embryo transition [55]. Further research on ceRNAs involved in reproductive regulation will be carried out in the turtle. Considering knowledge of the regulatory mechanism of gonadal development is scarce in non-mammal animals, our results help to enrich the understanding of the reproductive regulatory network in non-mammalian vertebrates.

## Conclusions

In the present study, we identified mRNAs, miRNAs, and lncRNAs using high-throughout sequencing data from the ovaries and testes of Chinese soft-shelled turtles and constructed the associated ceRNA networks. We identified 11,319 DEmRNAs, 633 DEmiRNAs, and 10,495 DElncRNAs in the ovary and testis. Furthermore, we constructed ceRNA networks, which included DEmRNAs, DEmiRNAs, and DElncRNAs that regulated the reproduction of the turtle. The present study provides an invaluable resource for further studies on the molecular regulation of reproduction in turtles.

## Methods

### Sample collection and RNA isolation

All investigations in the present study were performed according to the Animal Experimental Guidelines of the Ethical Committee of the University of China. Nine adult female turtles (body weight 600 ± 45 g, mean ± SD) and nine male turtles (body weight 750 ± 50 g) were obtained from Chenyuan Aquaculture Co., Ltd. of Xinyang, China, which were aged 24 months and cultured in the same pond. Samples were collected according to Experimental Animal Management Ordinance (Ministry of Science and Technology, 2004). To minimize suffering of the turtle, each turtle was euthanized with an overdose of 2 ml anaesthetic (2-phenoxyethanol; Sigma-Aldrich) by intraperitoneal administration and sacrificed by cervical dislocation before their reproductive season. The testes and ovaries were obtained after slaughtering and immediately stored at − 80 °C. Total RNA was isolated from each gonadal sample using TRIzol reagent (Invitrogen, USA). RNA concentration and quality were determined using Nanodrop, Qubit 2.0 and an Agilent 2100 bioanalyzer. The higher-quality RNA (the value of RIN ranged from 7.3 to 7.7) was stored at − 80 °C for library construction. The male and female turtles were divided into three groups, and the RNA from the three turtles was pooled. Six miRNA and lncRNA libraries from testes (*n* = 3) and ovaries (*n =* 3) were constructed.

### Library preparation and sequencing

MiRNA sequencing libraries were constructed using 2.5 ng of RNA per gonadal sample of turtles. The library was constructed following the manufacturer’s instructions for the NEB Next Ultra Small RNA Sample Library Prep Kit for Illumina (NEB, USA). Briefly, a 3′ SR adaptor was ligated by 3′ ligation enzyme mix, and the SR RT primer was used to prevent adaptor dimer formation. After that, the 5′ SR adaptor was ligated, and reverse transcription was performed to synthesise the first strand. Then, target fragments were amplified by RT-PCR using synthesised first-strand cDNA as the template, and the library was isolated and constructed by polyacrylamide gel electrophoresis. The library was assessed with an Agilent 2100 Bioanalyzer. Clustering of the index-coded samples was performed on a cBot Cluster Generation System using TruSeq PE Cluster Kit v4-cBot-HS (Illumina, USA) according to the manufacturer’s instructions. After that, the prepared libraries were sequenced on an Illumina HiSeq X Ten platform.

Sequencing libraries of mRNA and lncRNA were constructed using 1.5 μg of RNA per gonadal sample from which rRNA was removed by the Ribo-Zero rRNA Removal Kit (Epicentre, Madison, WI, USA). The libraries for sequencing were constructed using the NEBNext^R^ Ultra™ Directional RNA Library Prep Kit (NEB, Ipswich, MA, USA) for IlluminaR (NEB, USA) following the manufacturer’s recommendations. Briefly, first-strand cDNA and second-strand cDNA synthesis and the library fragments was purified by AMPure XP Beads (Beckman Coulter, Beverly, MA, USA). After that, the fragments that were 150–200 base pairs (bp) in length were selected. The strands containing U bases were removed by USER Enzyme (NEB, USA). PCR was then performed, and the library quality was evaluated on an Agilent Bioanalyser 2100. TruSeq PE Cluster Kitv3-cBot-HS (Illumina) was used for clustering on the acBot Cluster Generation System according to the manufacturer’s instructions. The library preparations were then sequenced on an Illumina HiSeq X Ten platform, and paired-end reads were generated.

### Quality control

Quality control of the reads was performed using FastQC (http://www.bioinformatics.babraham.ac.uk/projects/fastqc/). The raw data were first processed by in-house Perl scripts. Clean reads were obtained by discarding reads containing adapter and poly-N and low-quality reads. The Q20, Q30 and GC contents of the filtered reads were calculated and used for further analysis.

### Transcriptome assembly

The clean reads were mapped to the turtle genome v1.0 (PRJNA221645, NCBI) using HISAT2 [56] and Bowtie software [57]. The mapped reads of each sample were assembled using String Tie [58] according to the reference-based approach [59]. For the identified miRNAs, the assembled transcripts were compared with ncRNAs (rRNA, tRAN, snRNA, snoRNA, and other ncRNAs) and repeats using Bowtie software [57].

### Prediction of miRNA and lncRNA targets

The mapped reads were aligned to miRbase (http://www.mirbase.org/) [60] using miRDeep2 [61]. The characteristic hairpin structure of the miRNA precursors was used to predict novel miRNA. miRDeep2 [61] and Mfold software were used for predicting the structure of the unannotated miRNAs and their precursors.

After mapping the genome, the transcripts were screened for mRNA and lncRNAs. The transcripts with more than 200 nucleotides or those that had more than one exon were selected as lncRNA candidates. The candidate lncRNAs were further screened by CPC/CNCI/Pfam/CPAT, which could distinguish the protein-coding genes from the non-coding genes.

To annotate the functions of the lncRNAs, we predicted the target protein-coding genes of lncRNAs in *cis* and *trans*. The protein-coding genes ranging from 100 kb upstream to 100 kb downstream of a lncRNA were identified as *cis*-acting target genes. The genes that overlapped with the lncRNAs predicted by LncTar [62] were annotated as *trans*-acting target genes.

### Expression analysis

The expression levels of miRNAs in each sample were calculated and normalised using transcripts per million. The expression levels of protein-coding genes and lncRNAs were calculated as fragments per kilo base of exon per million fragments mapped and assessed using Cuffdiff (v2.1.1) [63]. Differentially expressed genes (DEGs) analysis of the miRNAs, lncRNAs, and mRNAs was performed using the DEGseq package (1.10.1) [64]. A false discovery rate (FDR) ≤ 0.01 and an absolute value of log2 (fold change, FC) ≥ 1 were determined for DEmiRNAs, DEmRNAs and DElncRNAs.

### Target gene prediction between miRNA and lncRNA

The prediction of interactions between miRNA and lncRNA was performed using MiRanda. Target genes of DEmiRNAs were predicted using MiRanda [65] and RNAhybrid [66].

### Bioinformatics analysis

To predict the functions of the miRNAs and lncRNAs, the target genes and differentially expressed genes were annotated against the NCBI non-redundant protein database (Nr), the Gene Ontology (GO) database, Kyoto Encyclopedia of Genes and Genomes (KEGG), and clusters of orthologous groups of proteins. GO terms with KS ≤0.05 and pathways with corrected *P* ≤ 0.05 were defined as significantly enriched.

### Quantitative real-time PCR analysis of miRNA and lncRNA

In the present study, 8 miRNAs and 7 lncRNAs were verified by using quantitative real-time PCR (qRT-PCR). The primers for miRNA and lncRNA are shown in Additional file 13. Total RNA of ovaries and testis was extracted by TRIzol reagent (Invitrogen, USA) strictly followed the manufacturer’s instructions. Using total RNA from the ovaries and testes of turtles as a template, first-strand cDNAs of miRNAs were obtained with a Mir-X miRNA first strand synthesis kit (Clontech, USA). Expression profiles of miRNAs were examined by SYBR qRT-PCR (Clontech). All reactions were performed in a CFX96 Touch™ instrument (Bio-Rad, USA).

All experiments were repeated at least three times. The results of the qRT-PCR data are presented as the mean ± standard error of the mean value. Statistical analyses were performed using the SPSS 16.0 software program (SPSS Inc., Chicago, IL, USA). Differences were considered statistically significant at *P* < 0.05.

### CeRNA network construction

To construct the lncRNA–miRNA–mRNA network, we predicted lncRNAs that might act as endogenous sponges based on lncRNAs that were up- or down-regulated by FC > 2.0 and *P* < 0.05, which significantly correlated with the miRNA predicted target genes. LncRNAs possessed miRNA response elements as predicted by RNA22 (http://cm.jefferson.edu/rna22/Precomputed) and PITA (http://genie.weizmann.ac.il/pubs/mir07/mir07_data.html). Furthermore, we calculated the Pearson correlation coefficient between two genes, and correlations (> 0.8) were selected to construct the networks. The networks were constructed by Cytoscape software [67].

## Supplementary information

**Additional file 1 **The results of reads mapped to the genome of *Pelodiscus sinensis*.

**Additional file 2.** The differentially expressed miRNAs between ovaries and testes

**Additional file 3.** The predicted families of DEmiRNAs

**Additional file 4.** The DEmRNAs between ovaries and testes

**Additional file 5.** The DElncRNAs between ovaries and testes

**Additional file 6.** Gene Ontology (GO) analysis of DEmiRNAs

**Additional file 7 **Gene Ontology (GO) analysis of DElncRNAs in *cis*

**Additional file 8 **Gene Ontology (GO) analysis of DElncRNAs in *trans*

**Additional file 9.** KEGG pathway analysis of DEmiRNAs

**Additional file 10 **KEGG pathway analysis of DElncRNAs in *trans*

**Additional file 11 **KEGG pathway analysis of DElncRNAs in *cis*

**Additional file 12.** ceRNA network results

**Additional file 13.** Primers used in qRT-PCR

## Data Availability

The datasets generated and/or analysed during the current study are available in the NCBI Sequence Read Archive (SRA) repository (accession number is PRJNA623141). The genome information of the turtle was obtained from NCBI (accession number is PRJNA221645). For the identified miRNAs, the assembled transcripts were compared with Silva database (https://www.arb-silva.de/), GtRNAdb database (http://gtrnadb.ucsc.edu/), and Rfam database (http://rfam.xfam.org/). The mapped reads of miRNAs were aligned to miRbase (http://www.mirbase.org/).

## Abbreviations

miRNA: MicroRNA
lncRNA: Long non-coding RNA
mRNA: messenger RNA
GO: Gene ontology
KEGG: Kyoto encyclopedia of genes and genomes
qRT-PCR: Quantitative real time polymerase chain reaction
DEmiRNA: Differentially expressed miRNA
DElncRNA: Differentially expressed lncRNA
DEmRNA: Differentially expressed mRNA
ceRNA: Compete endogenous RNA
