# Supplementary figures and images for: Genome-wide identification and comparison of differentially expressed profiles of miRNAs and lncRNAs with associated ceRNA networks in the gonads of Chinese soft-shelled turtle, Pelodiscus sinensis
Source: BMC Genomics. 2020 Jun 29;21:443. doi: 10.1186/s12864-020-06826-1 (PMC7322844; doi:10.1186/s12864-020-06826-1)

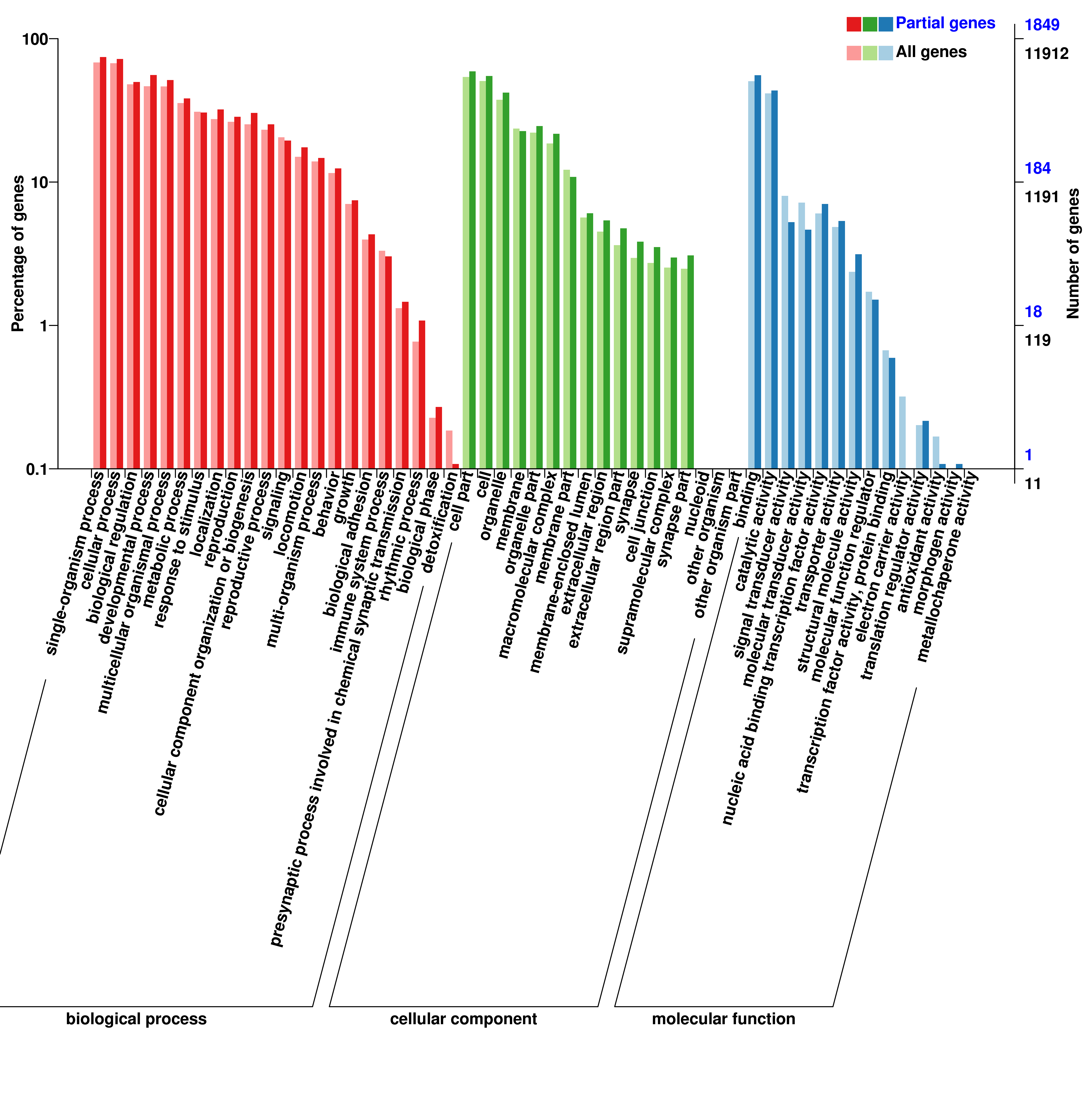

Supplement: Supplementary file 6 — Additional file 6. Gene Ontology (GO) analysis of DEmiRNAs [file 12864_2020_6826_MOESM6_ESM.png]

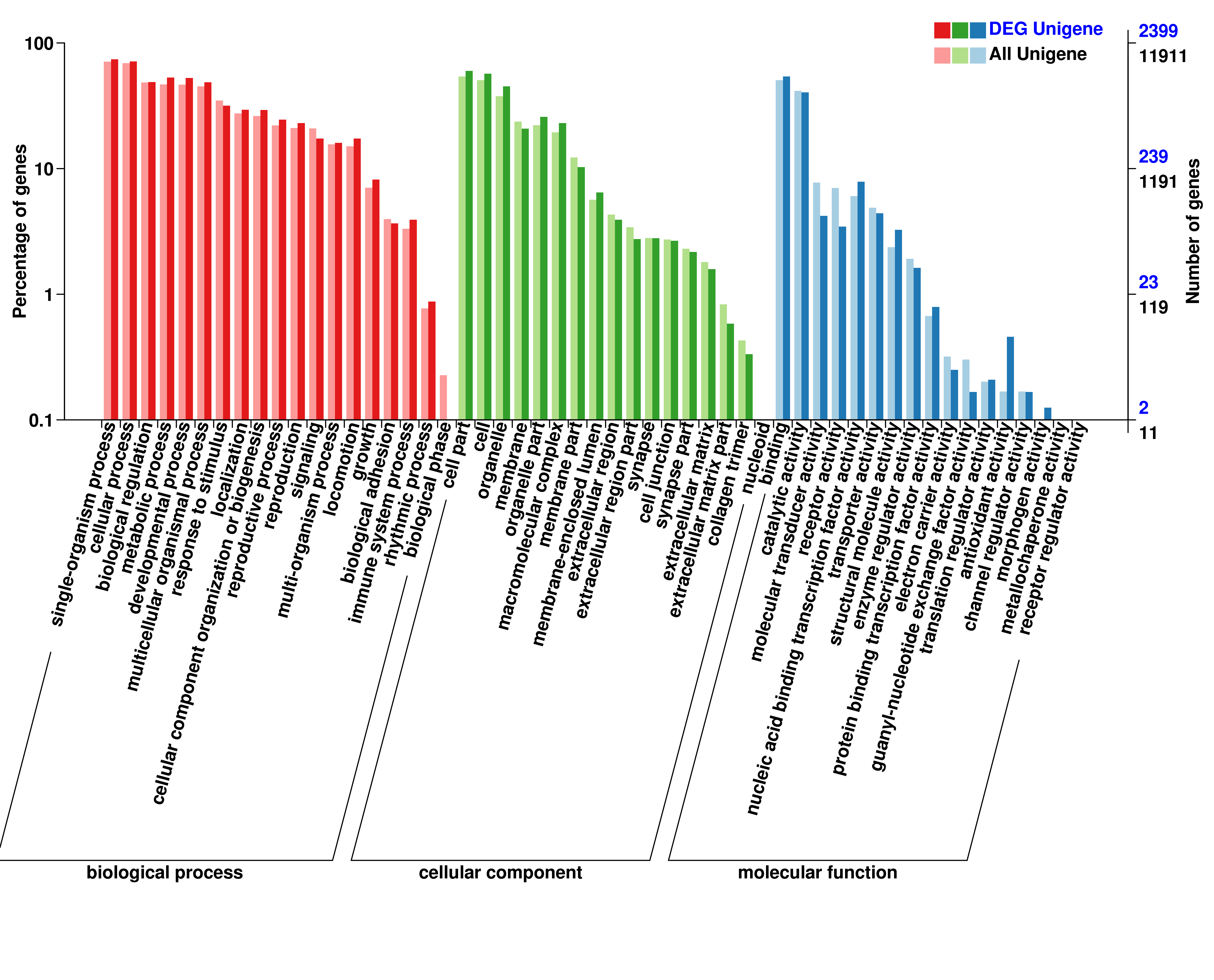

Supplement: Supplementary file 7 — Additional file 7 Gene Ontology (GO) analysis of DElncRNAs in cis [file 12864_2020_6826_MOESM7_ESM.png]
